# Convergence on an Optimal Way to Swim

**DOI:** 10.1371/journal.pbio.1002124

**Published:** 2015-04-28

**Authors:** Robin Meadows

**Affiliations:** Freelance Science Writer, Fairfield, California, United States of America

## Abstract

A study of eight independent instances of evolution of a distinct mode of swimming by animals such as rays and cuttlefish shows repeated arrival at a single optimal solution.

What do cuttlefish, skates, and bony fish have in common? Duh—they all swim! True, but it turns out they also share something that's not nearly so obvious. Despite the tremendous variation in body type and swimming stroke amongst these aquatic animals, their fins all have a mechanical property that maximizes speed, according to new research by Malcolm MacIver, Neelesh Patankar, and colleagues in this issue of *PLOS Biology*.

The team discovered that when these diverse animals swim, the undulations of their fins reflect an underlying constant. The specific wavelength—or ratio of undulation wavelength to the average amplitude of the undulations along the length of the fin—is essentially fixed at 20.

This property holds for 22 species scattered across the animal kingdom in three phyla (flatworms, mollusks, and chordates). Importantly, each of the species swims by keeping its body still while undulating elongated fins. These fins vary widely by species. For example, knifefish have single fins down the middle of their belly or back, triggerfish have paired fins on the top and bottom of their bodies, and cuttlefish have paired fins on the left and right. Called median/paired fin swimming, this technique contrasts with that of fish like trout, which swim by moving their tails and the tail ends of their bodies.

To see what's so special about a specific wavelength of 20 for fin undulations in median/paired fin swimmers, the researchers tested a robot with a knifefish-like fin as well as a computer-simulated median fin (Fig 1). They found that the number of undulations it took to maximize thrust varied with parameters including fin length, height, and shape. However, when thrust was examined through the lens of the wavelength-to-amplitude ratio, or specific wavelength, all of this variation collapsed and maximum thrust occurred at one particular ratio—about 20—which the researchers accordingly call the optimal specific wavelength (OSW).

**Fig 1:**
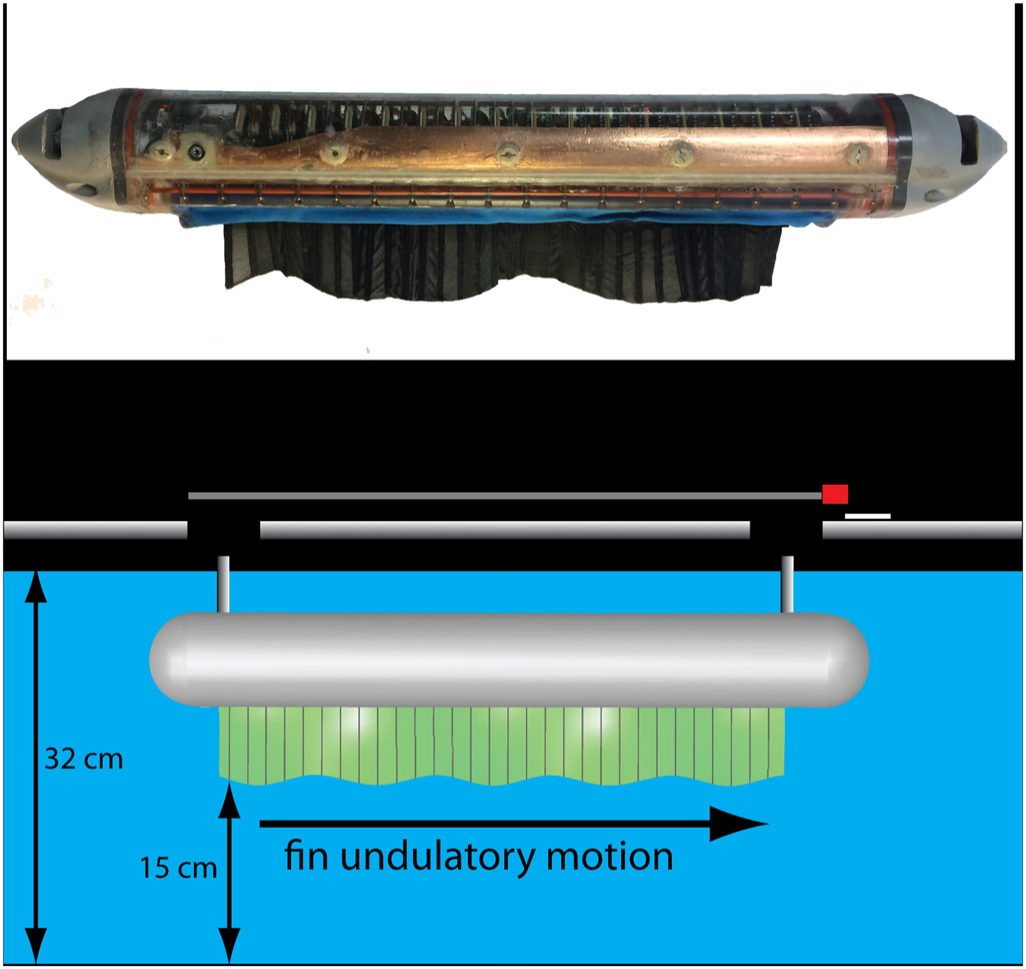
A robot helps understand biology: An underwater robot called Ghostbot was used, along with computer simulations, to test hypotheses concerning convergent evolution of an optimal movement pattern of elongated fins. *Image credit: Malcolm MacIver and Izaak Neveln*.

The researchers posit that the OSW may be at the sweet spot of two competing mechanisms in undulatory fins. In the "friction mechanism," steeply undulating fins move water backward more efficiently and so provide more thrust at a small specific wavelength. However, in the "velocity mechanism," shallowly undulating fins move water fast and so provide more thrust at a large specific wavelength. Because a fin cannot be both steep and shallow, both mechanisms obviously cannot be optimized simultaneously. Indeed, the researchers found that swimming slows when fins are too steep or too shallow. Rather, an intermediate steepness yields the intermediate specific wavelength that maximizes thrust, which of course is the OSW.

Many OSW swimmers have no known ancestor that swam with median/paired fins, and the 22 species in this analysis belong to eight clades (groups of organisms stemming from a common ancestor). This suggests that the OSW evolved independently at least eight times.

Such convergent evolution typically involves morphological features, such as wings in insects, birds, and mammals. In contrast, the OSW is a pattern of movement found in diverse fin types in disparate aquatic animals. But while rare, convergent evolution of a locomotory pattern that presents a mechanically optimal solution is not unprecedented. Notably, in tail-end swimmers like trout, propulsive efficiency is maximized at the Strouhal number, which accounts for swimming speed, lateral fin excursion, and the frequency of tail beating.

Tail-end swimmers dominate in jawed fishes, accounting for 97% of the 33,000 catalogued species. A likely reason for their preponderance is that although the OSW optimizes speed in median/paired fin swimming, tail-end swimming is much faster. This raises the question of why the slower OSW swimming has arisen repeatedly in species whose ancestors were tail-end swimmers.

The answer may be that median/paired fin swimming takes less energy, which offers its own advantages. In addition, some ecological conditions may favor OSW swimming. The researchers speculate that this may be so for South American knifefish, which live in murky water and are nocturnal. They navigate this dark environment via electric fields generated in the trunks of their bodies, and these fields would be distorted if swimming entailed moving their trunks instead of keeping them OSW-style rigid.

This elegant work reveals that a physical problem—how to get from here to there—can be optimized by a wondrous diversity of biological solutions. Moreover, these findings strengthen the case that mechanical optimization can drive evolution, contributing to the longstanding debate over the evolutionary roles of randomness versus physical constraints that limit the solutions that are feasible in living creatures. As the researchers point out, quantifying physical properties that underlie biological phenomena could help us recognize when an optimal mechanical solution is likely to drive convergent evolution.
